# Acoustic Bubble and Magnetic Actuation-Based Microrobot for Enhanced Multiphase Drug Delivery Efficiency

**DOI:** 10.3390/mi14122169

**Published:** 2023-11-29

**Authors:** Jihyeok Park, Youngkwang Kim, Jinwon Jeong, Deasung Jang, Daegeun Kim, Sangkug Chung

**Affiliations:** 1 Department of Mechanical Engineering, Myongji University, Yongin 17058, Republic of Korea; wlgurgg@mju.ac.kr (J.P.); ygkim@mju.ac.kr (Y.K.); 2 Department of Electrical and Computer Engineering, Baylor University, Waco, TX 76706, USA; jinwon_jeong1@baylor.edu; 3 Department of Mechanical Engineering, The University of British Columbia, Vancouver, BC V6T 1Z4, Canada; jang.deasung@ubc.ca; 4 Microsystems, Inc., Yongin 17058, Republic of Korea

**Keywords:** microrobot, acoustic bubble, target drug delivery technology, cavitational microstreaming, magnetic liquid metal

## Abstract

This paper proposes an acoustic bubble and magnetic actuation-based microrobot for enhancing multiphase drug delivery efficiency. The proposed device can encapsulate multiphase drugs, including liquids, using the two bubbles embedded within the microtube. Additionally, using the magnetic actuation of the loaded magnetic liquid metal, it can deliver drugs to target cells. This study visualized the flow patterns generated by the oscillating bubble within the tube to validate the drug release principle. In addition, to investigate the effect of the oscillation properties of the inner bubble on drug release, the oscillation amplitude of the inner bubble was measured under various experimental variables using a high-speed camera. Subsequently, we designed a microrobot capable of encapsulating bubbles, drugs, and magnetic liquid metal and fabricated it using microfabrication technology based on ultra-precision 3D printing. As a proof of concept, we demonstrated the transport and drug release of the microrobot encapsulating the drug in a Y-shaped channel simulating a blood vessel. The proposed device is anticipated to enhance the efficiency of drug therapy by minimizing drug side effects, reducing drug administration frequency, and improving the stability of the drug within the body. This paper is expected to be applicable not only to targeted drug delivery but also to various biomedical fields, such as minimally invasive surgery and cell manipulation, by effectively delivering multiphase drugs using the simple structure of a microrobot.

## 1. Introduction

According to the World Health Organization (WHO), cancer is one of the leading causes of mortality globally [1,2]. Treatments for these cancers include surgical treatment for the direct removal of cancer cells as well as chemotherapy for drug administration to treat the cancer cells [3,4]. Among them, chemotherapy is widely used for cancer treatment because it administers drugs through oral and intravenous injections, allowing quick drug delivery to all organs through the bloodstream [5,6]. However, while cancer treatment with chemotherapy can effectively remove cancer cells, the drugs administered can also affect healthy cells, causing side effects such as hair loss, loss of appetite, and emesis [7]. Recently, there has been a great deal of research into targeted drug therapy to reduce the side effects of chemotherapy and increase the effectiveness of treatment [8,9,10]. These therapies can increase absorption by preventing premature degradation of the drug while minimizing the impact on healthy cells by focusing the drug on target cells [11,12,13,14]. As a way to improve the efficiency of targeted drug therapy, targeted drug delivery technologies using microrobots is gaining prominence [15].

Targeted drug delivery technology using microrobots utilizes various control systems to precisely control drugs through untethered manipulation even in the viscous environment of the body, enabling effective delivery of drugs to target cells through minimally invasive surgery [16,17,18]. As a result, many research groups are continuing their investigations into microrobots for targeted drug delivery. The primary research areas for microrobots include developing methods to effectively propel and locomote them to the target sites using various energy-conversion mechanisms as well as ensuring the efficient delivery of therapeutic agents to the target cells [19].

Firstly, in the field of microrobotic propulsion research, many research groups are investigating various propulsion techniques based on chemical reactions, biology, and principles such as acoustic wave and magnetic fields [20,21]. Among these techniques, propulsion methods using magnetic fields are gaining attention in biomedical applications due to their ability to precisely control microrobots remotely and penetrate biological tissues without causing adverse reactions [22,23]. The working principle of a magnetic field is applied in two main ways: a magnetic gradient force using a gradient magnetic field and magnetic torque using a uniform magnetic field [24]. Conventional propulsion methods for microrobots have utilized magnetic torque to align magnetic materials and the magnetic gradient force for propulsion [25]. Yesin et al. and Jeong et al. conducted research using a propulsion method that combined uniform and gradient magnetic fields generated by a pair of Helmholtz and Maxwell coils, achieving precise control of microrobots in 2D and 3D space, respectively [26,27]. However, the magnetic gradient force was proportional to the size of the microrobot, so a strong magnetic gradient force was required to apply this at the microscale [28]. High electric currents to generate strong magnetic gradient forces result in a great deal of joule heat, which has the potential to negatively affect biological tissue, making it unsuitable for biomedical applications [29]. For the above reasons, a propulsion method with a helical mechanism that generates a translational corkscrew movement within a magnetic torque-based rotating magnetic field has been developed, which has advantages for propulsion in a low Reynolds number of viscous environments in the body [30,31]. Honda et al. introduced rotating magnetic-field-based helical wires followed by a propulsion method that uses artificial helical flagella, which was inspired by the flagellar motion of bacteria in Zhang et al. [32,33]. However, the propulsion method using a rotating magnetic field merely induced a simple rotational motion in the microrobots; hence, the development of an auxiliary helical mechanism is essential to achieve propulsion and to convert rotational motion into translational motion.

Secondly, in the field of drug manipulation, many research groups are investigating methods for efficient drug control to target cells, leveraging mechanisms such as magnetic actuation, environmentally responsive polymers, and gaseous bubbles. Geo et al. proposed a nanowire-based microrobot that delivers loaded drugs to target cells using the magnetic attraction of polymeric particles fabricated by encapsulating Ni segments and magnetic nanoparticles [34]. The proposed microrobot demonstrated drug delivery by capturing drug-loaded polymeric particles using magnetic actuation and delivering them to targeted cancer cells. For drug delivery, methods using polymer-based stimuli-responsive hydrogels can rapidly change their properties in response to shifts in temperature or pH, enabling applications in new fields of targeted drug delivery technologies [35,36,37]. Li et al. proposed a magnetically propelled, pH-responsive microgripper that integrated pH-sensitive hydrogels with magnetic actuation for drug control [38]. The proposed microgripper utilized trapping and unfolding motions in pH-varying solutions to deliver drugs to target cells. However, such drug delivery mechanisms have limitations in that they cannot control liquid-phase drugs, which have a higher penetration rate than solid-phase drugs in an aqueous medium due to the difficulty in completely sealing the drug. Hence, Jeong et al. proposed a microrobot utilizing gaseous bubbles for the control of liquid-phase drugs and conducted research on controlling the encapsulated drug to target cells using selective acoustic actuation [39]. However, this study utilized a dual-channel structure that separated the microtube for drug delivery from the one for drug diffusion, resulting in a larger size of the robot, and it did not include research results on viscosity to reflect the actual blood environment.

**Figure 1:**
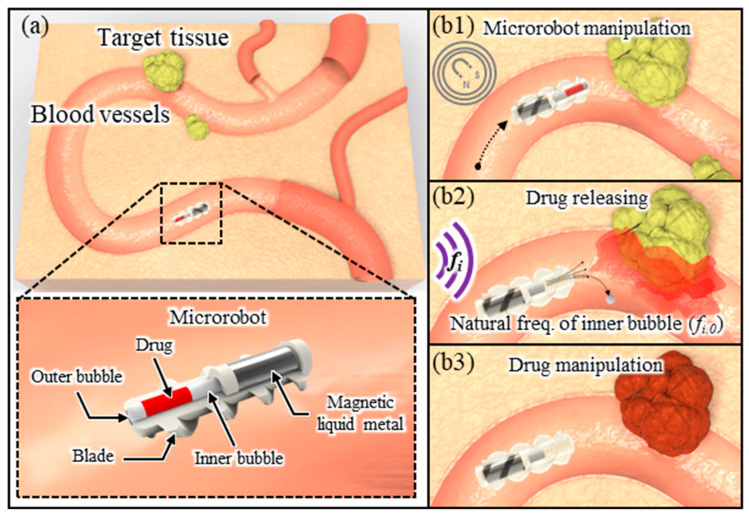
Schematic diagram of the proposed targeted drug delivery technology: (**a**) test setup and designed microrobot; (**b1**–**b3**) drug manipulation (carrying and releasing) using acoustic excitation of the inner bubble at natural frequency.

This paper presents a microrobot using a magnetic liquid metal for transportation to target cells and acoustic bubbles for efficient drug manipulation. We present a novel propulsion mechanism that harnesses the magnetic liquid metal’s deformable properties, enabling efficient microrobot movement in diverse environments. Furthermore, we demonstrate the wireless manipulation of multiphase drugs with varying physical properties in an aqueous medium using only gaseous bubbles, eliminating the need for complex mechanical mechanisms. This study presents a novel method for delivering multiphase drugs by analyzing the oscillation properties of acoustically excited bubbles within the microrobot under various experimental variables. The proposed microrobot consists of a microtube, a magnetic liquid metal carrier, and a mounted blade on the body of the microrobot, as shown in Figure 1a. Within the microtube, drugs are encapsulated using two bubbles of different volumes (an inner and outer bubble, respectively). For magnetic actuation, the magnetic liquid metal is loaded into the liquid metal carrier. Upon applying a magnetic field to the magnetic liquid metal, the microrobot encapsulating the drug is transported to the target cells (Figure 1(b1)). After the drug is transported to the target tissues, applying an acoustic wave of a natural frequency to an inner bubble embedded within the microtube causes the inner bubble to oscillate, generating a microstreaming flow (Figure 1(b2)). Through this process, the outer bubble covering the entrance of the tube is removed, allowing the encapsulated drug to be released from the microtube and delivered to the target tissues (Figure 1(b3)). This paper is expected to be applicable not only to targeted drug delivery but also to various biomedical fields, such as minimally invasive surgery and cell manipulation, by effectively delivering multiphase drugs using a simple structure of microrobot.

## 2. Principle

The following describes the operating principle of the proposed device. When a hydrophobic microtube with one end sealed is submerged in an aqueous medium, a column-shaped gaseous bubble is automatically trapped inside. When the gaseous bubble trapped inside the microtube is acoustically excited, the bubble oscillates (expands and contracts) due to its compressibility [40]. During the oscillation of the acoustic bubble, the meniscus of the bubble periodically moves within the microtube, and this movement generates intersecting flow patterns at the entrance of the microtube. According to fluid mechanics, if the Reynolds number (Re=ρUL/μ) is not too small, two different flow patterns occur at the entrance to the microtube [41]. Here, *ρ* is the density of the fluid, *U* is the flow velocity, and *L* is a characteristic dimension that typically represents the diameter of the bubble. One of the flow patterns is an inflowing omnidirectional flow, while the other is an outflowing unidirectional jet; these two patterns alternately form at the entrance of the microtube. Consequently, the two asymmetric flow patterns merge into a singular jet flow (microstreaming flow) due to the high momentum flux [42,43]. The acoustically excited gaseous bubble inside a microtube with one end sealed behaves as a damped harmonic oscillator [44,45,46]. The natural frequency of a bubble is approximately given as [46]:

(1) $$f_{0}=\frac{1}{2\pi }\sqrt{\frac{\kappa P_{0}}{\rho L_{0}L_{B}}}$$

The natural frequency of a bubble is approximately 1 ≤ *κ* ≤ γ, where γ (1.4 for air) *κ* is the specific heat ratio of the gas in the bubble. Here, κ is determined to be 1.2 based on the studies by Chen and Prosperetti [44]. $P_{0}$ is the pressure at steady state, ρ is the density of the liquid medium, $L_{0}$ is the length of the liquid filling the microtube, and $L_{B}$ is the length of the bubble within the microtube. The integrated equation with these variables indicates that the natural frequency of the bubble is a function dependent on the length of the bubble, $L_{B}$. Additionally, the bubble inside the tube can achieve its maximum oscillation amplitude at its natural frequency, and the intensity of the microstreaming flow is simultaneously maximized.

## 3. Results and Discussion

### 3.1. Experiment Setup and Methods

**Figure 2:**
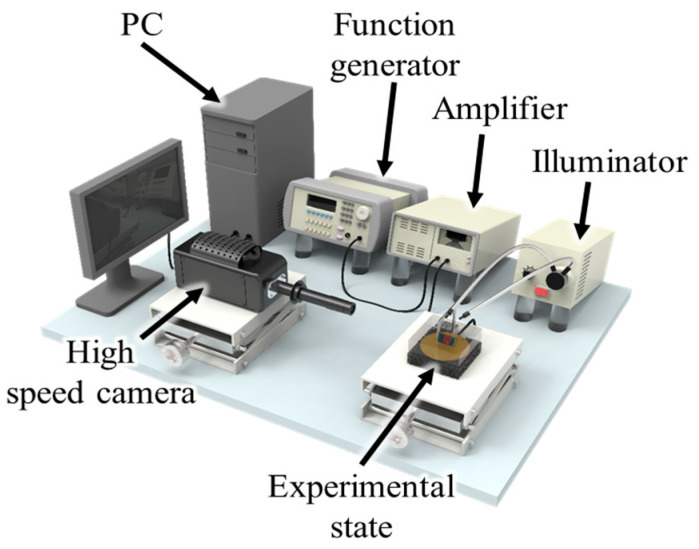
Schematic of the experimental setup.

Figure 2 shows the experimental setup for analyzing the oscillation properties of the acoustically excited bubble. A disk-shaped piezoactuator (MFT-27T 4.1A1, KEPO Co., Ningbo, China) was used for the acoustic excitation, and a function generator (33210A, Agilent Co., Santa Clara, CA, USA) and an amplifier (PZD700, Trek Co., Lockport, NY, USA) were used to apply an alternating current. The piezoactuator was attached to the bottom of the chamber (8 (L) × 4.5 (W) × 2.5 (H) cm^3^) using an adhesive (3M, CAT. No. 237). The oscillation properties of the bubble were observed through a zoom lens (VZM^TM^ 450io, Edmund Optics., Barrington, NJ, USA) mounted on a high-speed camera (Phantom Micro eX4, Vision Research Inc., Wayne, NJ, USA).

**Figure 3:**
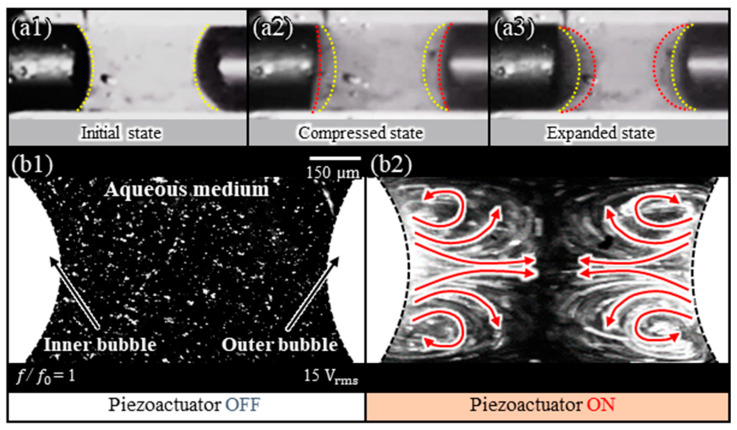
(**a1**–**a3**) Sequential snapshots of the oscillations of two bubbles (inner and outer) generated by the acoustically excited inner bubble within a glass tube; (**b1**,**b2**) microstreaming flow visualization of two bubbles oscillated by an inner bubble within a glass tube.

In Figure 3, we visualize the flow patterns generated by the oscillating bubble within the tube to validate the proposed drug release principle. To observe the flow within the tube, a glass tube with high permeability (length: 4 mm) was used; to increase the stability of the bubble, parylene-C was deposited on the inside surface using chemical vapor deposition (CVD). The fluid mixed with fluorescent particles (8 μm dia., FF1015-01, Fluostar, EBM Co., Tokyo, Japan) was injected into the middle spot of a sealed-end glass tube using a microsyringe to form an inner bubble (length: 2 mm). Subsequently, the glass tube was submerged in an aqueous medium to automatically form an outer bubble separating the inner fluid from the aqueous medium. To observe the oscillation of the bubble within the glass tube, an acoustic wave was applied to the inner bubble (applied voltage: 15 V_rms_; frequency: 1.1 kHz). At this time, the applied acoustic wave corresponded to the natural frequency of the inner bubble. Figure 3(a1–a3) sequentially show the initial state, compressed state, and expanded state of a bubble oscillated by an acoustic wave.

Next, we visualized the microstreaming flow induced by bubble oscillations within the glass tube (Figure 3(b1,b2)). To visualize the microstreaming flow, we illuminated the fluorescent particles using a 532-nm-laser source (MGL H-532 nm, Changchun New Industries Optoelectronics Tech Co., Changchun, China). Based on the experimental results, a violent microstreaming flow was observed within the fluid between the inner bubble and the outer bubble due to the oscillations of the acoustically excited inner bubble. Furthermore, the microstreaming flow generated by the two bubbles could interact to push the outer bubble out of the tube.

### 3.2. Experimental Variables: Bubble Length

**Figure 4:**
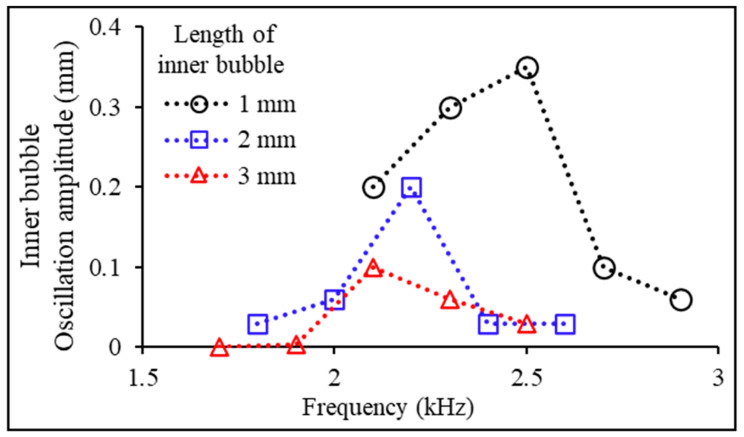
Measurement of oscillation amplitudes of the acoustically excited inner bubbles (1, 2, and 3 mm lengths) at various acoustic frequencies for drug release and outer bubble removal.

This study investigated the oscillation properties of the inner bubble relative to its length within a microrobot to examine the effect of the oscillation amplitude of the inner bubble on drug release. Firstly, the oscillation amplitude of the inner bubble was measured at different acoustic frequencies using three different lengths (1 mm, 2 mm, and 3 mm) of the inner bubble as variables (Figure 4). Here, oscillation amplitude refers to the difference in length between the maximum expansion and compression states of the bubble. In this experiment, the natural frequency of the bubble was derived from the point at which the bubble exhibited its maximum oscillation amplitude. For the experiment, a hydrophobic Teflon tube (diameter: 400 μm) was used. A liquid drug solution (0.03 wt %, safranin solution) was injected into the middle spot of the tube using a microsyringe to generate an inner bubble. During this process, the drug was injected into the microtube, leaving a gap of 0.3 mm from its entrance. Subsequently, the Teflon tube was submerged in an aqueous medium to form an outer bubble. In the experiment depicted in Figure 4, the length of the outer bubble was consistently maintained at 0.3 mm. The experimental results revealed that as the length of the inner bubble increased, both the oscillation amplitude and the natural frequency of the inner bubble decreased. The maximum oscillation amplitude of the inner bubble was observed when its length was 1 mm. At this time, the frequency of the applied acoustic wave matched the natural frequency of the inner bubble ($f_{Inner,0}$ = 2.5 kHz).

**Figure 5:**
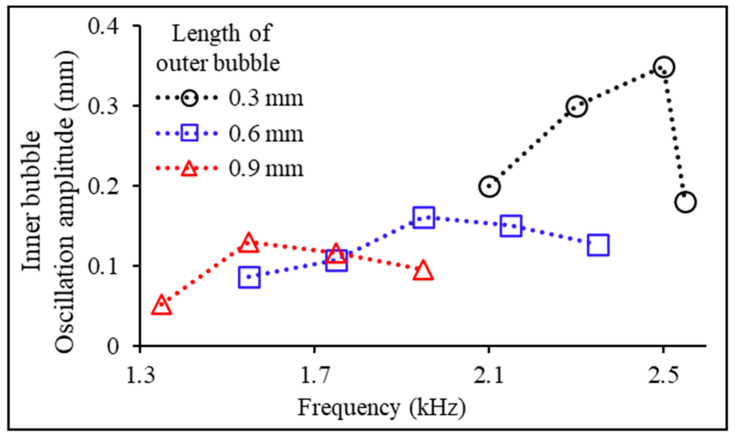
Measurement of the oscillation amplitude of the acoustically excited inner bubble depending on the lengths of outer bubbles (0.3, 0.6, and 0.9 mm) at various acoustic frequencies for drug release and outer bubble removal.

Secondly, the oscillation amplitude of the inner bubble was measured at different acoustic frequencies using three different lengths (0.3 mm, 0.6 mm, and 0.9 mm) of the outer bubble as variables (Figure 5). We used the same fabrication method as in our previous experiments. Through previous experiments, the length of the inner bubble was consistently maintained at 1 mm, where it exhibited the maximum oscillation amplitude at its natural frequency. The experimental results indicated that as the length of the outer bubble increased, both the oscillation amplitude and the natural frequency of the inner bubble decreased. The maximum oscillation amplitude of the outer bubble was observed when its length was 0.3 mm. At this time, the frequency of the applied acoustic wave matched the natural frequency of the inner bubble ($f_{Inner,0}\;$= 2.5 kHz).

### 3.3. Experimental Variables: Experimental Fluid Viscosity

**Figure 6:**
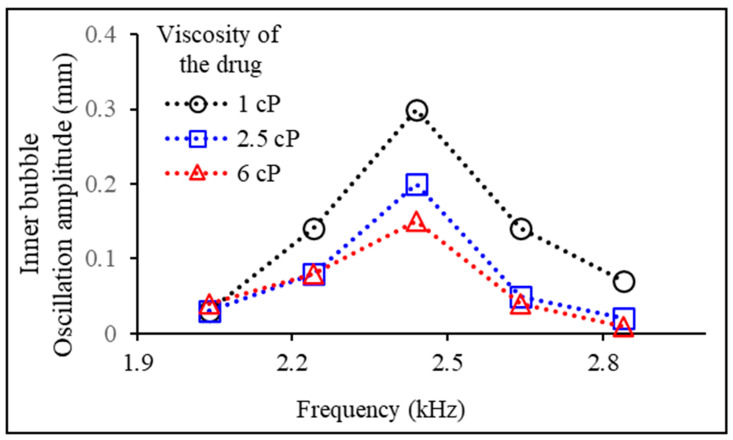
Measurement of oscillation amplitudes of the acoustically excited inner bubbles depending on the viscosity of the drug (1, 2.5, and 6 cP) at various acoustic frequencies.

To investigate the impact of experimental fluid viscosity on drug release, we examined the oscillation properties of the inner bubble while varying the drug viscosity and the surrounding medium. Figure 6 shows the effect of the viscosity of a drug encapsulated in two bubbles in a microtube on the oscillation properties of the inner bubble. The oscillation amplitude of the inner bubble was measured for drugs with three different viscosities (1 cP, 2.5 cP, and 6 cP) across various acoustic frequencies. In the experiment shown in Figure 6, the viscosity of the medium surrounding the microtube was consistently maintained at 1 cP. Based on the experimental results, while the increase in drug viscosity did not affect the natural frequency of the inner bubble, a decreasing trend in the oscillation amplitude of the inner bubble was observed.

**Figure 7:**
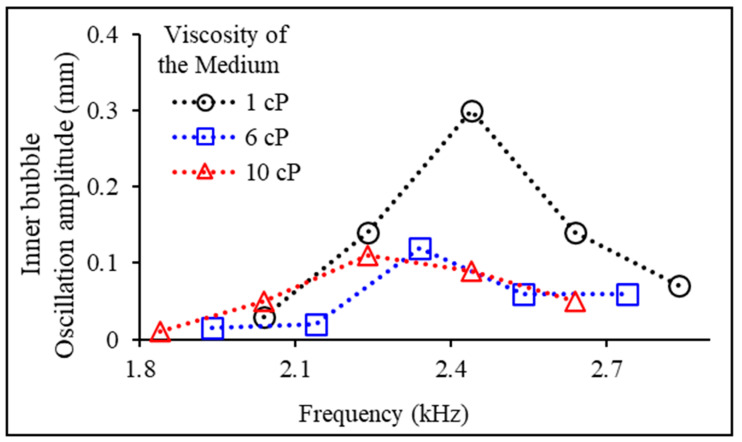
Measurement of oscillation amplitudes of the acoustically excited inner bubbles depended on the viscosity of the medium surrounding the microtube (1, 6, and 10 cP) at various acoustic frequencies.

Figure 7 shows the effect of the viscosity of the medium surrounding the microtube on the oscillation properties of the inner bubble. The oscillation amplitude of the inner bubble was measured for medium with three different viscosities (1, 6, and 10 cP) across various acoustic frequencies. In the experiment in Figure 7, the viscosity of the drug inside the robot was consistently maintained at 1 cP. Based on the experimental results, as the viscosity of the medium surrounding the microtube increased, a decrease in both the natural frequency and the oscillation amplitude of the inner bubble was observed. As a result, as the viscosity of the experimental fluid increased, the amplitude of inner bubble oscillation decreased, thus experimentally confirming the difficulties associated with drug release.

### 3.4. Multiphase Drug Release Experiments

**Figure 8:**
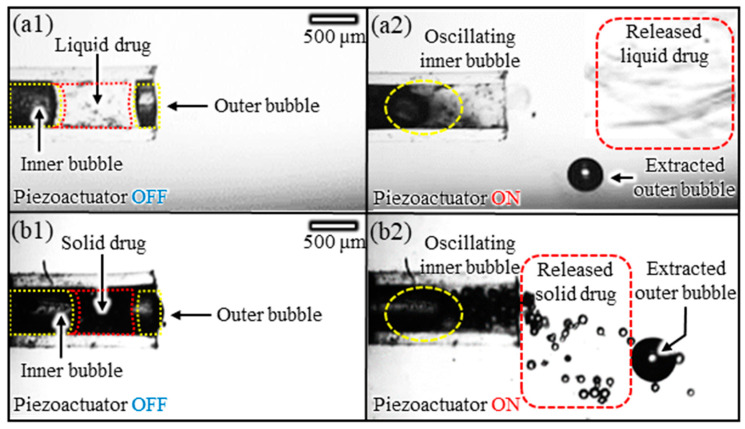
Sequential snapshots of drug release using acoustic excitation of inner bubble: (**a1**,**a2**) liquid-phase and (**b1**,**b2**) solid-phase drugs released by acoustic excitation of the inner bubbles.

Figure 8 shows the results of an experiment using acoustic bubbles to release drugs in multiphase (liquid and solid phases) with different physical properties. The inside of the Teflon tube (length: 2 mm; diameter: 400 μm) consisted of an inner bubble and an outer bubble (length: 1.0, 0.3 mm, respectively), with liquid- and solid-phase materials trapped between the two bubbles, respectively. For drug release, an acoustic wave corresponding to the natural frequency of the inner bubble was applied (2.5 kHz). For the liquid drug release experiments, we utilized a chemical reaction solution $\left(Pb{\left({NO}_{3}\right)}_{2}\right)$ as the liquid-phase drug and a KI solution as the medium surrounding the microtube. Upon applying an acoustic wave at the natural frequency to the inner bubble, it was observed that the encapsulated drug was released out of the microtube and underwent a chemical reaction ($Pb{\left({NO}_{3}\right)}_{2}+2KI\;\to \;{PbI}_{2}{+2KNO}_{3}$) with the medium surrounding the microtube (Figure 8(a1,a2)). In the solid drug release experiments, we used glass beads (70 μm dia, CAT. No 5212, Sigmund Lindner Co., Warmensteinach, Germany) as the solid-phase drug. We confirmed the sealing of solid drugs using bubbles and the release of solid drugs utilizing the oscillation properties of the bubbles (Figure 8(b1,b2)). As a result, by successfully demonstrating the release of multiphase drugs, we experimentally verified the drug delivery capability utilizing the superior opening and closing abilities of the bubble.

### 3.5. Design and Fabrication of Microrobots

**Figure 9:**
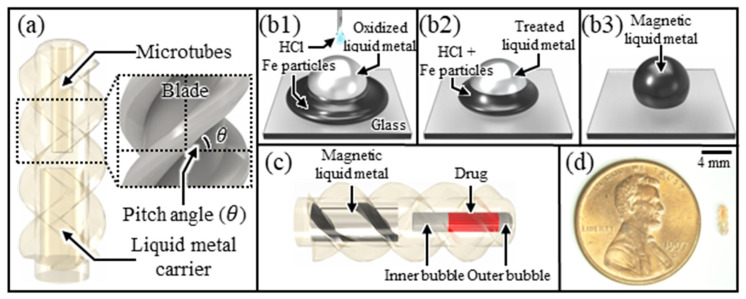
Design and fabrication microrobots: (**a**) design of a microrobot with microtubes, a liquid metal carrier, and blades; (**b1**–**b3**) conceptual diagram of using Fe particles and HCl solution to create a magnetic liquid metal; (**c**) microrobot containing drugs and magnetic liquid metal; (**d**) fabricated microrobot shown to scale.

The proposed microrobot was composed of several key components: a microtube (diameter: 400 μm, length: 2 mm) for drug encapsulation, a liquid metal carrier (diameter: 800 μm, length: 2 mm) to load the magnetic liquid metal, and a helical blade designed for propulsion (Figure 9). Based on prior experimental results, the microtube was designed with optimized lengths for the inner and outer bubbles (1.0 and 0.3 mm, respectively) to maximize drug release.

The propulsion of microrobots using rotating magnetic fields is advantageous for efficient propulsion in high-viscosity, low-Reynolds-number environments, and this is being utilized by many research groups [30,31,32,33]. For propulsion within a rotating magnetic field, the shape of the microrobot is designed to be helical, converting rotational motion into translational motion. The propulsion efficiency is maximized when the helical blade on the robot body has a pitch angle of 45° [47]. Therefore, in this study, we designed the blades on the microrobot’s body with reference to the results of previous research (Figure 9a). Next, a magnetic liquid metal was fabricated for the magnetic actuation of the microrobot. Gallium-based liquid metal simultaneously exhibits the properties of metal and the fluidity of a liquid, notably providing the potential for infinite shape deformation. However, it easily oxidizes in oxygen-rich environments and does not exhibit magnetic properties. To solve this problem, a magnetic liquid metal was fabricated that prevents oxidation and possesses magnetic properties. The magnetic liquid metal was fabricated by triggering the intercalation chemical reaction of liquid metal with Fe particles (diameter: 5 μm) and HCl solution (37 wt %) [48]. Through this process, a magnetic liquid metal coated with Fe particles on its surface was fabricated (Figure 9(b1–b3)). The fabricated magnetic liquid metal was loaded into the lower carrier of the microrobot, allowing the microrobot to be transported by a magnetic field (Figure 9c). Finally, Figure 9d shows the fabrication of the designed microrobot using ultra-precision 3D-printer-based microfabrication technology (microArch^TM^ S240, Jive Solutions Co., Hwasong, Republic of Korea). The microrobot was fabricated using medical-grade resin and deposited with parylene-C to enhance biocompatibility, making it suitable for various applications, including medical devices and the biomedical field [49,50].

**Figure 10:**
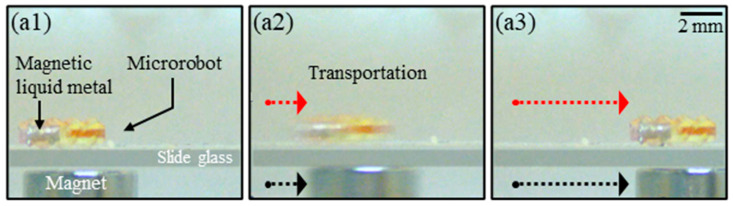
Time-lapse image of a vertical magnetic-field-based magnetic liquid metal actuation with a magnet placed under a slide glass showing the changing position of the microrobot; (**a1**–**a3**) horizontal Movement of the Fabricated Microrobot.

Figure 10 shows the experimental results to verify the magnetic actuation of the magnetic liquid metal. A circular-shaped permanent magnet (NdFeB) was used to manipulate the magnetic liquid metal using a magnetic field. In this study, the magnetic flux density applied to the microrobot was recorded at 300 gauss as measured with a gauss meter (Model SG-9115, Segye Scientific Co., Ltd., Seongnam, Republic of Korea). As the distance between the permanent magnet and the microrobot increased, the magnetic field density decreased, which tended to reduce the movement of the microrobot. A microrobot was placed on top of a slide glass (thickness: 0.1 mm) and a permanent magnet on the bottom, and the microrobot was moved horizontally via the movement of the magnet to verify the transport of the magnetic liquid metal by the magnetic force. In future studies, we plan to conduct investigations into wireless propulsion methods using electromagnetic forces and to perform transporting experiments of microrobots in experimental fluids with a viscosity similar to blood.

### 3.6. Experimental Demonstrations

**Figure 11:**
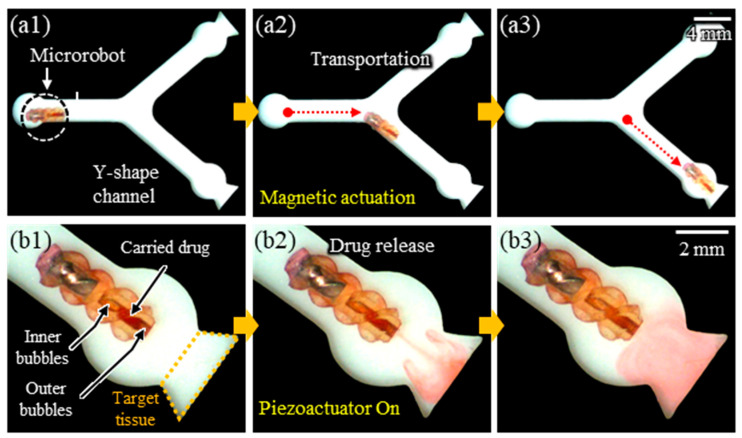
Sequential snapshots of the proposed targeted drug delivery technology: (**a1**–**a3**) magnetic actuation of a fabricated microrobot in a Y-shaped channel filled with water; (**b1**–**b3**) drug manipulation (carrying and releasing) by acoustically controlling bubbles embedded in the microrobot.

As a proof of concept, the proposed targeted drug delivery technology was demonstrated using the fabricated microrobot in a Y-shaped channel filled with water (Figure 11). The microrobot, loaded with magnetic liquid metal, was first transported to the target tissues through magnetic actuation (Figure 11(a1–a3)). Subsequently, an acoustic wave was applied to the inner bubble embedded in the microtube, and the outer bubble was pushed out by the oscillation of the inner bubble. This action released the drug. Following the drug release, the remaining inner bubble induced a microstreaming flow, leading to the diffusion of the drug (Figure 11(b1–b3)).

## 4. Conclusions

This study introduces a novel microrobot for multiphase drug delivery that combines acoustic bubbles and magnetic actuation to enhance efficiency. We experimentally validated magnetic actuation for transporting microrobots to target tissues and acoustic bubble actuation for effective drug delivery. To confirm the drug release principle, we visualized the flow patterns generated by the oscillating inner and outer bubbles within the microtube. As a result, drug release was confirmed due to the interaction between the two bubbles. We investigated the oscillation properties of the inner bubble within the microrobot while considering the physical properties of the embedded bubble and the experimental fluid. The results show that as the length of the bubble and the viscosity of the experimental fluid increased, the oscillation amplitude of the inner bubble decreased, experimentally confirming the difficulties of drug release. Experiments were conducted on the release of multiphase drugs with different physical properties using acoustic bubbles. The natural frequency of the inner bubble was applied for drug release, and the proposed technology was experimentally validated by demonstrating drug release in liquid ($Pb{\left({NO}_{3}\right)}_{2}$) and solid (glass beads) phases. Finally, we verified the magnetic actuation of a microrobot loaded with magnetic liquid metal by utilizing a microrobot fabricated through ultra-precision 3D-printing-based microfabrication technology. Subsequently, by acoustically exciting the inner bubble, we successfully released the encapsulated drug, providing experimental validation for the proposed targeted drug delivery technology. This innovative technology enables the simple control of various multiphase drugs (solid- and liquid-phase) using a straightforward microrobot structure. In addition, it can enhance drug delivery efficiency by utilizing a drug delivery method using microrobots with fewer side effects, in contrast to traditional methods such as oral or intravenous injection. The proposed technology holds promise not only for targeted drug delivery but also for applications across various biomedical fields, including minimally invasive surgery and cell manipulation.

## Data Availability

No new data were created or analyzed in this study. Data sharing is not applicable to this article.
